# Efficient filtration of fibrinogen solution by a virus removal filter

**DOI:** 10.1016/j.btre.2026.e00977

**Published:** 2026-08-25

**Authors:** Tomona Harada, Kumiko Michikawa, Mana Konno, Takeshi Yokoyama, Mikihiro Yunoki, Daisuke Miyamoto

**Affiliations:** a Technology Development Department, R&D and Business Development, Asahi Kasei Life Science Corporation, Miyazaki, Japan; b Research and Development Division, Japan Blood Products Organization, Tokyo, Japan

**Keywords:** Arginine, Fibrinogen, Prefiltration, Virus removal filter

## Abstract

Historically, achieving efficient filtration of fibrinogen solutions using virus removal filters has been challenging due to the fibrinogen’s tendency to aggregate and its molecular size and structure. In this study, the feasibility of fibrinogen using the filtration the Planova™ 20 N filter and its virus removal efficacy under optimized filtration conditions were investigated. Optimization of arginine (400 mM) and fibrinogen (2 mg/mL) concentration in the solution, along with pre-filtration to remove the aggregates, was essential for achieving efficient filtration. Notably, Planova 20 N filter exhibited robust virus removal performance and the biological activity of fibrinogen including its clotting function was preserved, indicating that protein integrity was maintained throughout the process.

## Highlights

• Optimized 400 mM arginine suppresses fibrinogen aggregation and improves filterability.
• Planova 35 N pre‑filtration enhances throughput and removes aggregates.
• Higher temperature up to 35 °C increases filtration throughput.
• Planova 20 N achieved ≥6.6 log_10_ porcine parvovirus removal under optimized conditions.
• Fibrinogen biological activity is preserved through filtration steps.

## 1 Introduction

Fibrinogen is a coagulation factor derived from plasma that is widely used in therapeutic applications. Prior to the development of solvent-detergent (SD) virus inactivation techniques in the 1980s, fibrinogen products carried the risk of transmitting hepatitis B [1]. SD treatment has demonstrated strong efficacy in inactivating enveloped viruses; however, it remains ineffective against non-enveloped viruses and does not provide comprehensive virus safety. To address this gap, additional virus inactivation methods such as heat treatment, UV-C irradiation, and chemical treatment have been introduced. These approaches offer broader virus inactivation efficacy, targeting both enveloped and non-enveloped viruses. However, each method carries specific limitations, including potential degradation of protein structure or loss of biological activity, incomplete virus inactivation, or operational complexity. Consequently, enhancing virus safety measures, particularly against non-enveloped viruses, remains a critical focus in the purification of fibrinogen solutions.

In contrast, virus removal filters offer effective virus removal without affecting the biological activity of the protein. The first virus removal filter was introduced in 1987, and by the 1990s, filters with a nominal pore size of 35 nm (Planova™ 35 N) were introduced into manufacturing processes. In 1993 and 2001, Planova™ 15 N and 20 N filters with smaller nominal pore size of 15 and 20 nm, respectively, were implemented. Effective removal of smaller viruses, including those with particle sizes around 20 nm, has been demonstrated with the use of these filters [[2], [3], [4]]. The combination of SD treatment and virus filtration with small pore size is a powerful measure against the viral contamination of fibrinogen or other biological products. However, integrating virus filtration into fibrinogen purification processes presents significant challenges. Fibrinogen is a large, elongated plasma protein with a major axis of approximately 45 nm and a minor axis of approximately 9 nm. This anisotropic structure may contribute to the difficulty of virus filtration. It is hypothesized that the passage through virus removal filter pores depends, at least in part, on molecular orientation: fibrinogen is unlikely to pass through the pore when its major axis is oriented across the pore entrance, whereas passage may occur when the molecule is aligned along its minor axis. Therefore, efficient filtration of fibrinogen requires process conditions that minimize aggregation and reduce pore blockage without compromising protein recovery or biological activity [[5], [6], [7], [8]].

Arginine is known to reduce protein aggregation by destabilizing misfolded and aggregated species through preferential interaction with protein surfaces [9]. Such reduction in intermolecular interactions has been shown to decrease particle formation that causes membrane fouling during virus removal filtration [10]. Previous studies have demonstrated that the addition of basic amino acids, including arginine and lysine, can improve the filterability of fibrinogen solutions [5,11]. Currently, at least five fibrinogen products have been approved worldwide and at least two of these include a virus filtration step [12,13]. Notably, both of these products—Fibrinogen HT I.V. 1 g “JB” and Fibryga®—contain L-arginine hydrochloride. These reports and product compositions supported our selection of arginine as a formulation component for improving fibrinogen filterability.

In this study, we investigated the optimal conditions for introducing the use of virus removal filters into fibrinogen product processes with a focus on the suppression of flux decay when using Planova 20 N filters by the addition of arginine.

## 2 Materials and methods

### 2.1 Materials

Sodium chloride (catalog no. 191-01665), citric acid (catalog no. 030-05525), sodium hydroxide (catalog no. 192-02175) and L(+)-arginine hydrochloride (catalog no. 018-04625) were purchased from Fujifilm-Wako, Tokyo, Japan. Commercially available Fibrinogen HT I.V. 1 g "JB" (Japan Blood Products Organization, Tokyo, Japan) was used in experiments. Porcine parvovirus (PPV) NADL-2 (ATCC VR-742) and PK13 cells (ATCC CRL-6489) from ATCC, Manassas, VA, USA were used. Pre-filtration and virus filtration were conducted using Millex-GP (0.22 μm pore size, catalog no SLGPR33RB) and Millex-VV (0.1 μm pore size, catalog no SLVVR33RS) from Merck Millipore (Bedford, MA, USA) and Planova 20 N (0.0001 m^2^, prepared for the research purposes) and 35 N (0.001 m^2^) (Asahi Kasei Life Science Corporation, Tokyo, Japan). Measurement of biological activity was conducted using thrombin reagent LQ (catalog no.13360), Standard Human Plasma (catalog no. 13430) and TC Buffer (catalog no. 13460) from Sysmex, Kobe, Japan and PD Midi-Trap G-25 columns (catalog no. 28918008) from Cytiva, Marlborough, MA, USA.

### 2.2 Preparation of fibrinogen solutions

Each vial of Fibrinogen HT I.V. 1 g “JB” was dissolved in the provided 50 mL of water for injection to produce a 20 mg/mL fibrinogen solution. These solutions were pre-filtered using Millex-GP followed by Millex-VV pre-filters at room temperature and 0.5 bar to remove impurities in the solutions. Citric acid was added to reach 50 mM, NaCl was added to reach 150 mM, and the pH was adjusted to 7.

### 2.3 Preparation of fibrinogen solution spiked with PPV

PPV purified by density gradient ultracentrifugation was spiked into the prepared fibrinogen solution to a titer 7.75 log_10_TCID_50_/mL. To remove aggregated virus particles, pre-filtration was performed using Planova 35 N at 0.5 bar.

### 2.4 Filtration by Planova 20N

Fibrinogen solutions were subjected to constant pressure filtration at 25, 30 or 35 °C for 3 h at 1 bar.

### 2.5 Virus infectivity assay

Virus titer in the feed and filtrate samples was determined by the 50% tissue culture infectious dose (TCID_50_) method. Briefly, the samples were serially diluted 10-fold and inoculated to the PK13 cells in 96-well plates. After incubation for 10 days, the infected wells were determined by hemagglutination (HA) [14]. TCID_50_ was calculated using the Spearman-Kärber method [15], and the detection limit was 0.73 log_10_ TCID_50_/mL.

### 2.6 Measurement of fibrinogen concentration

Fibrinogen concentration was determined by UV absorbance (Synergy H1, BioTek, VT, USA). Fibrinogen solutions were dispensed in 100 μL aliquots a 96-well plate, and absorbance at 280 nm was measured. Fibrinogen recovery rate was calculated from the ratio of solution concentration before and after filtration.

### 2.7 Measurement of viscosity

Viscosity was determined by a vibratory viscometer (VM-10A, Sekonic, Tokyo, Japan). The measurement conditions were a sample volume of 20 mL and an equilibration time of 10 min.

### 2.8 Measurement of dynamic light scattering (DLS)

DLS measurements were performed using a Malvern DLS system (Zetasizer Nano ZSP, Malvern Panalytical, Malvern, UK). The measurement conditions were as follows: sample volume, 80 μL; equilibration time, 120 s; equilibration temperature, 25 °C; number of measurements, 3; and detection angle, 173° backscatter. Data analysis was performed using the cumulants method.

### 2.9 Measurement of biological activity

Fibrinogen activity was determined according to Clauss [16] using thrombin reagent LQ on a KC4 Delta Coagulometer (Tcoag, Wicklow, Ireland). Calibration was done with Standard Human Pasma. Buffer exchange of fibrinogen samples into TC Buffer was performed using PD Midi-Trap G-25 columns.

## 3 Results and discussion

### 3.1 Effect of arginine concentration on fibrinogen throughput

**Fig. 1:**
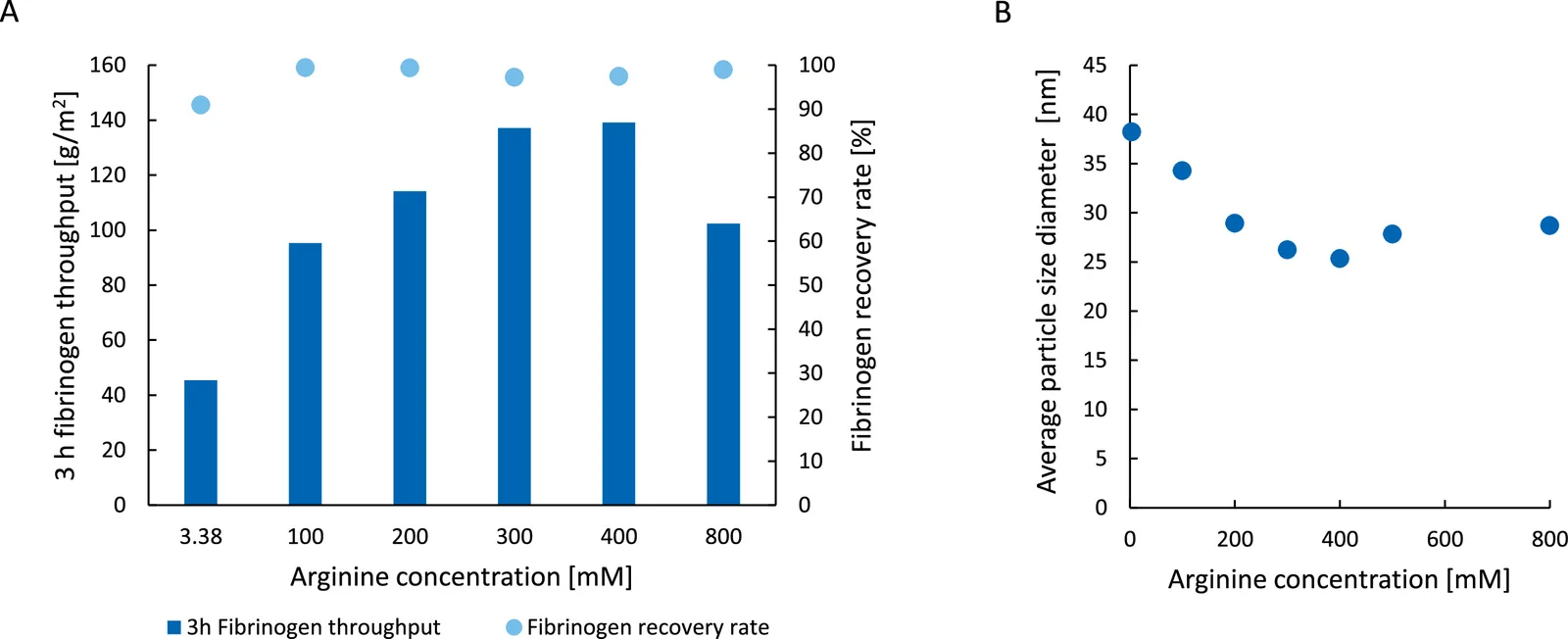
**Fibrinogen filterability and average particle size characterization at different arginine concentrations.** (A) Effect of arginine concentration on 3 h fibrinogen throughput and fibrinogen recovery rate. (B) Effect of arginine on average particle size of fibrinogen.

The optimal arginine concentration for virus filtration of 1 mg/mL fibrinogen in 50 mM citric acid, 150 mM NaCl, pH 7 on Planova 20 N filters was evaluated based on 3 h fibrinogen throughput and fibrinogen recovery rate (Fig. 1A). Fibrinogen throughput increased with increasing arginine concentration up to 400 mM but decreased slightly for arginine concentration in excess of 400 mM. Fibrinogen recovery was high between 94.7% to 99.5% for arginine concentrations between 100 and 800 mM and lower (91.0%) at the lowest arginine concentration of 3.38 mM. Characterization of average particle size of fibrinogen in arginine solution measured by DLS shows decreasing average particle size to 400 mM arginine and a slight increase in size above that concentration (Fig. 1B).

These results indicate that arginine may form associations with fibrinogen that reduce the average particle size as measured by DLS [17] and consequently result in improved filtration throughput. It should be noted that DLS does not directly measure the physical dimensions of particles but rather determines the hydrodynamic diameter from the translational diffusion coefficient through the Stokes-Einstein relationship.

It should also be noted that fibrinogen is an elongated molecule with a major axis of approximately 45 nm, whereas DLS reports a hydrodynamic diameter derived from diffusional behavior in solutions. Therefore, the DLS-derived particle size should not be interpreted as a direct measure of the molecular length of fibrinogen.

In previous work by Zydney and co-workers, the observed decline in performance of virus removal filters with increasing antibody concentration was shown to be correlated with increased average particle size measured by DLS [18]. Likewise, our DLS results are in agreement with the observation that average particle size changes as a function of arginine concentration.

In a study of the renaturation of recombinant Fab fragments, Rudolph and Buchner demonstrated that the addition of arginine preferentially destabilizes misfolded or aggregated species, thereby promoting correct folding which prevents aggregation and stabilizes the structure [9]. The relative renaturation of Fab fragments increased with increasing arginine concentration, reaching a maximum at 400 mM. Beyond this concentration, further increases in arginine led to a decrease in relative renaturation. While arginine stabilizes protein structures, there is an optimal concentration for its effectiveness.

Fibrinogen is known to be susceptible to self-association and aggregation under solution stresses such as elevated protein concentration, temperature changes, and interfacial stress [19,13].

Such aggregation or formation of higher-order species can increase the apparent hydrodynamic diameter and promote pore blockage during virus filtration. Therefore, the decrease in the DLS-derived average particle size observed at appropriate arginine concentrations is consistent with reduced self-association or aggregation of fibrinogen, which in turn may contribute to improved filtration throughput.

With the arginine concentration fixed at 400 mM, the effects of citric acid and sodium chloride concentrations on fibrinogen throughput were examined. No significant differences in fibrinogen throughput were observed for citric acid concentrations between 10 and 50 mM or sodium chloride concentrations between 50 and 300 mM (data not shown). Subsequent filtration experiments were conducted with fibrinogen in 400 mM arginine, 50 mM citric acid, and 150 mM sodium chloride.

### 3.2 Effect of temperature on fibrinogen throughput

**Fig. 2:**
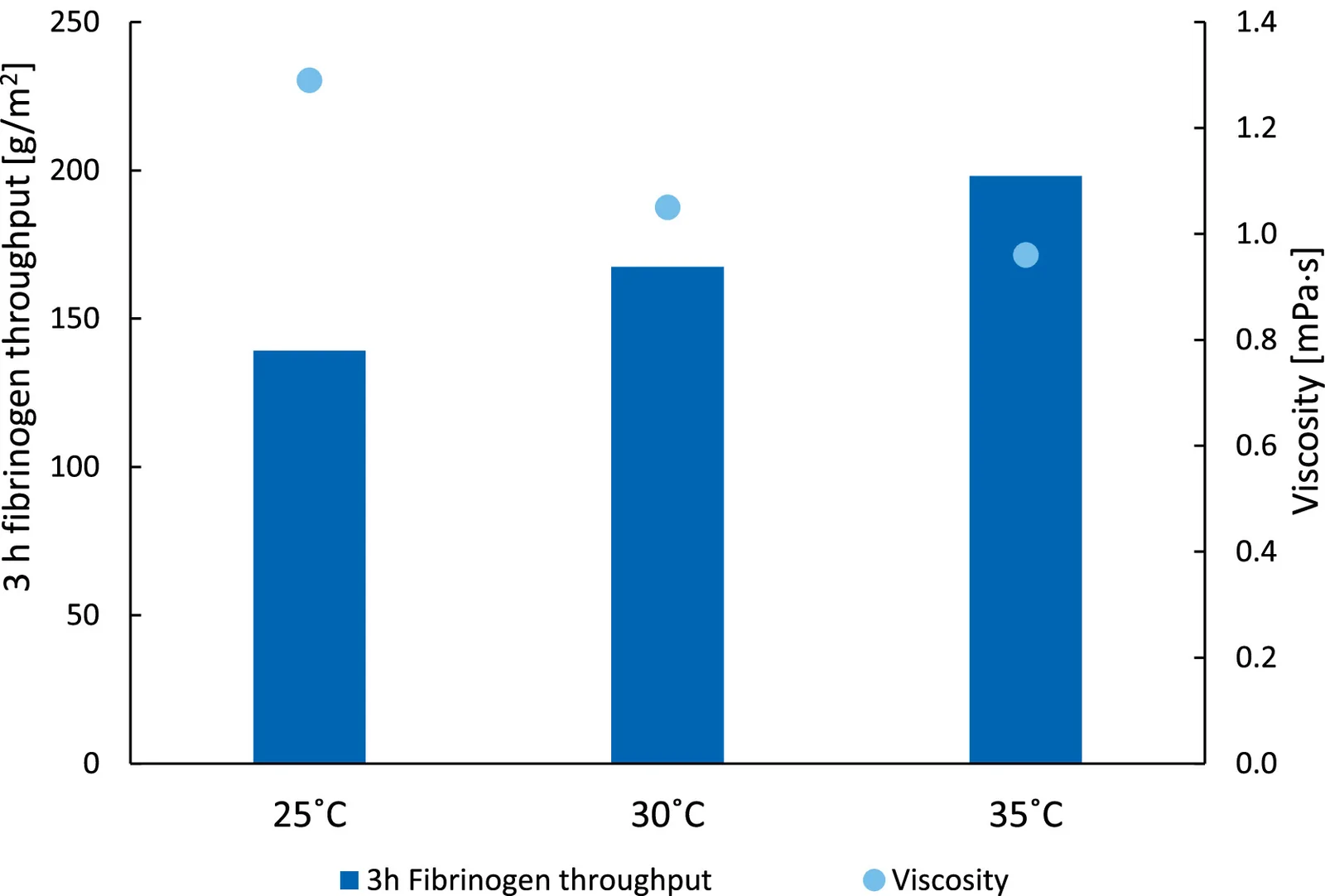
Effect of temperature on 3 h fibrinogen throughput and viscosity.

The optimal temperature for virus filtration was investigated using 1 mg/mL fibrinogen in 50 mM citric acid, 400 mM arginine, 150 mM NaCl, pH 7. Fibrinogen throughput increased with increasing filtration temperature from 25 to 35 °C and was inversely proportional to viscosity (Fig. 2). According to Hagen-Poiseuille's equation, flux and the amount of fibrinogen throughput correlate with viscosity.

Although the improvement in throughput with increasing temperature was primarily consistent with the decrease in solution viscosity, possible effects of temperature on the structural state of fibrinogen should also be considered. In this study, filtration was limited to 35 °C or lower because fibrinogen is known to be susceptible to thermal denaturation at higher temperatures. Therefore, the improved throughput observed at 35 °C is likely attributable mainly to the decrease in viscosity rather than to denaturation or major structural alteration of fibrinogen [19].

### 3.3 Effect of fibrinogen concentration on throughput

**Fig. 3:**
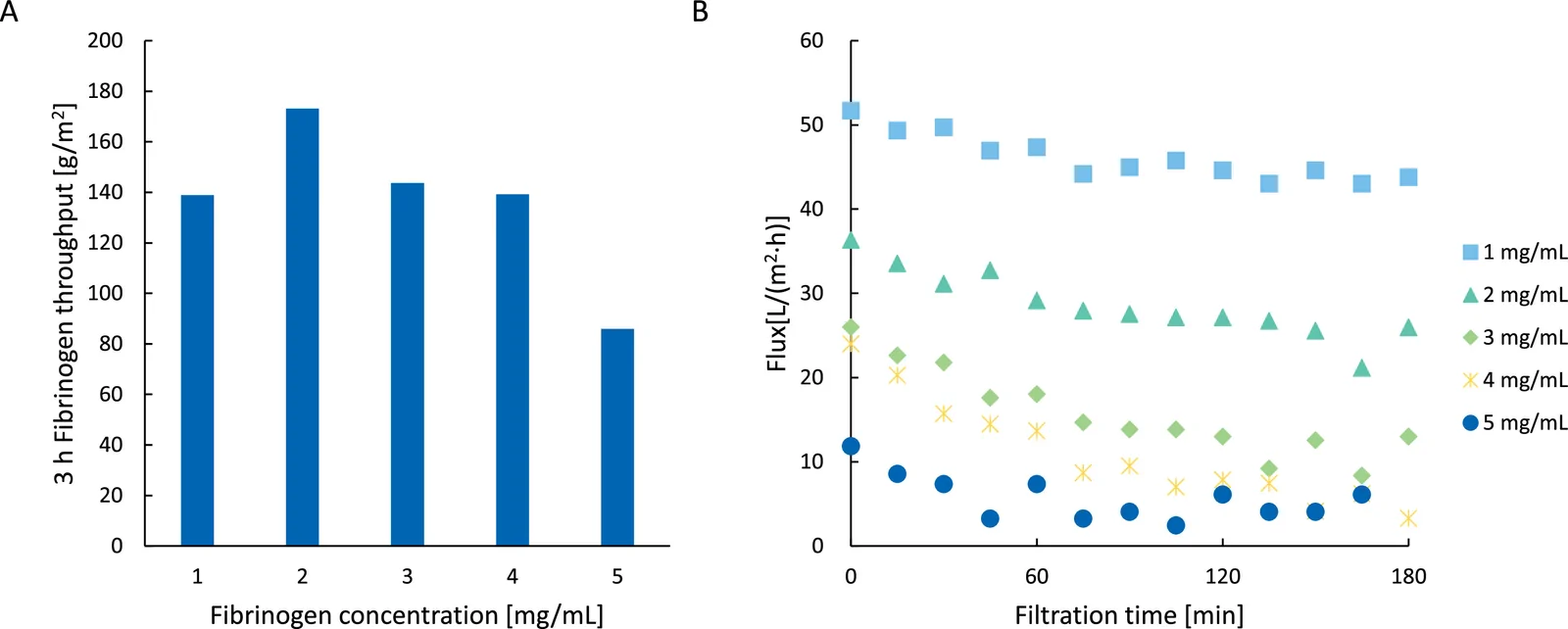
Filterability at different fibrinogen concentrations. (A) Effect of fibrinogen concentration on 3 h fibrinogen throughput. (B) Fibrinogen flux profiles for all tested fibrinogen concentrations.

The optimal fibrinogen concentration for virus filtration was investigated using 1 to 5 mg/mL fibrinogen in 50 mM citric acid, 400 mM arginine, 150 mM NaCl, pH 7 with filtration at 25 °C. The maximum 3 h fibrinogen throughput was achieved for 2 mg/mL fibrinogen (Fig. 3A). According to flux profiles for each fibrinogen concentration, the initial flux decreased with increasing fibrinogen concentration (Fig. 3B), and average particle size and viscosity increased with increasing fibrinogen concentration (Table 1).

**Table 1:** Average particle size and viscosity for fibrinogen at different concentrations.

Fibrinogen concentration [mg/mL]	Average particle size d. [nm]	Viscosity [mPaཥs]
0	-	1.26
1	25.36	1.28
2	28.43	1.30
3	28.27	1.32
4	28.48	1.35
5	31.41	1.40

The diffusion interaction parameter, k_D_, which quantifies the attractive or repulsive intermolecular forces, and is correlated with the osmotic second virial coefficient, B_2_, was demonstrated to be suitable for characterizing the properties of drug substances in solution [20,21].

The measured kD value of fibrinogen in the presence of 3.38 mM arginine was −0.0108 L/g, whereas the kD value increased to −0.00711 L/g in the presence of 400 mM arginine. This result indicates that arginine reduced the attractive intermolecular interactions between fibrinogen molecules. Zydney and co-workers investigated the filterability of virus removal filters using proteins with different kD values [10]. Their study demonstrated that the difference between kD values of −0.0089 and −0.0044 L/g affects protein filtration performance, and these results were consistent with those of the present study. Accordingly, the increase in the kD value and the resulting reduction in fibrinogen self-association were considered to be related to the improved filtration throughput observed in the presence of arginine.

This kD value (−0.00711 L/g) indicates that arginine effectively suppresses fibrinogen aggregation, although it does not promote its dispersion. Consequently, as the fibrinogen concentration increased, the average particle size and viscosity of the solution also increased, leading to a further decrease in flux.

### 3.4 Effect of pre-filtration on fibrinogen throughput and biological activity

**Fig. 4:**
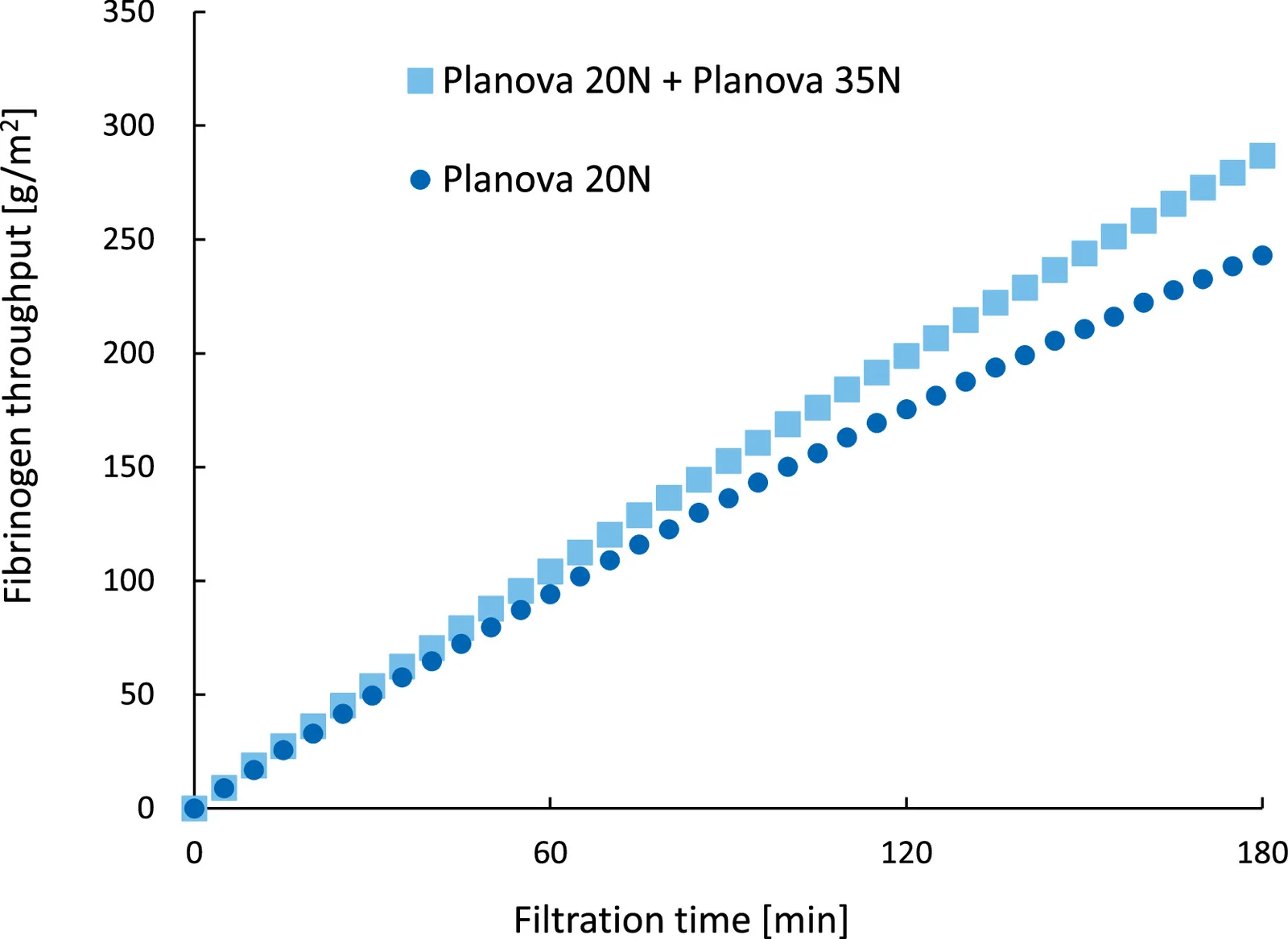
Effect of Planova 35 N pre-filtration on fibrinogen throughput.

The effectiveness of pre-filtration of fibrinogen solution with a Planova 35 N filter was confirmed for 2 mg/mL fibrinogen in 50 mM citric acid, 400 mM arginine, 150 mM NaCl, pH of 7 with filtration at 35 °C. Using fibrinogen solution filtered with a 0.1 µm size removal filter, virus removal filtration was performed on a Planova 20 N without and with additional pre-filtration was performed using a Planova 35 N filter. Fibrinogen throughput was improved by using Planova 35 N as a pre-filter (Fig. 4), owing to its effective removal of high molecular weight substances (Table 2). Although Planova 75 N has traditionally been employed as a pre-filter due to its larger pore size, the Planova 35 N filter with a smaller pore size was shown here to serve as an effective pre-filter [22,23].

**Table 2:** Average particle size of fibrinogen after pre-filtration with different pre-filters.

Pre-filter	Average particle size d. [nm]
Millex-VV (0.1 μm)	28.43
Planova 35N	27.44

The biological activity of fibrinogen was maintained either the pre-filtration with Planova 35 N filter or virus filtration with the Planova 20 N filter (Table 3).

**Table 3:** Biological activity of each fibrinogen solution.

Process step	Fibrinogen biological activity Clauss / Total fibrinogen [mg/mg]
Before filtration	0.76
After pre-filtration with Planova 35N	0.76
After filtration with Planova 20N	0.73

### 3.5 Viral clearance filtration with PPV

**Fig. 5:**
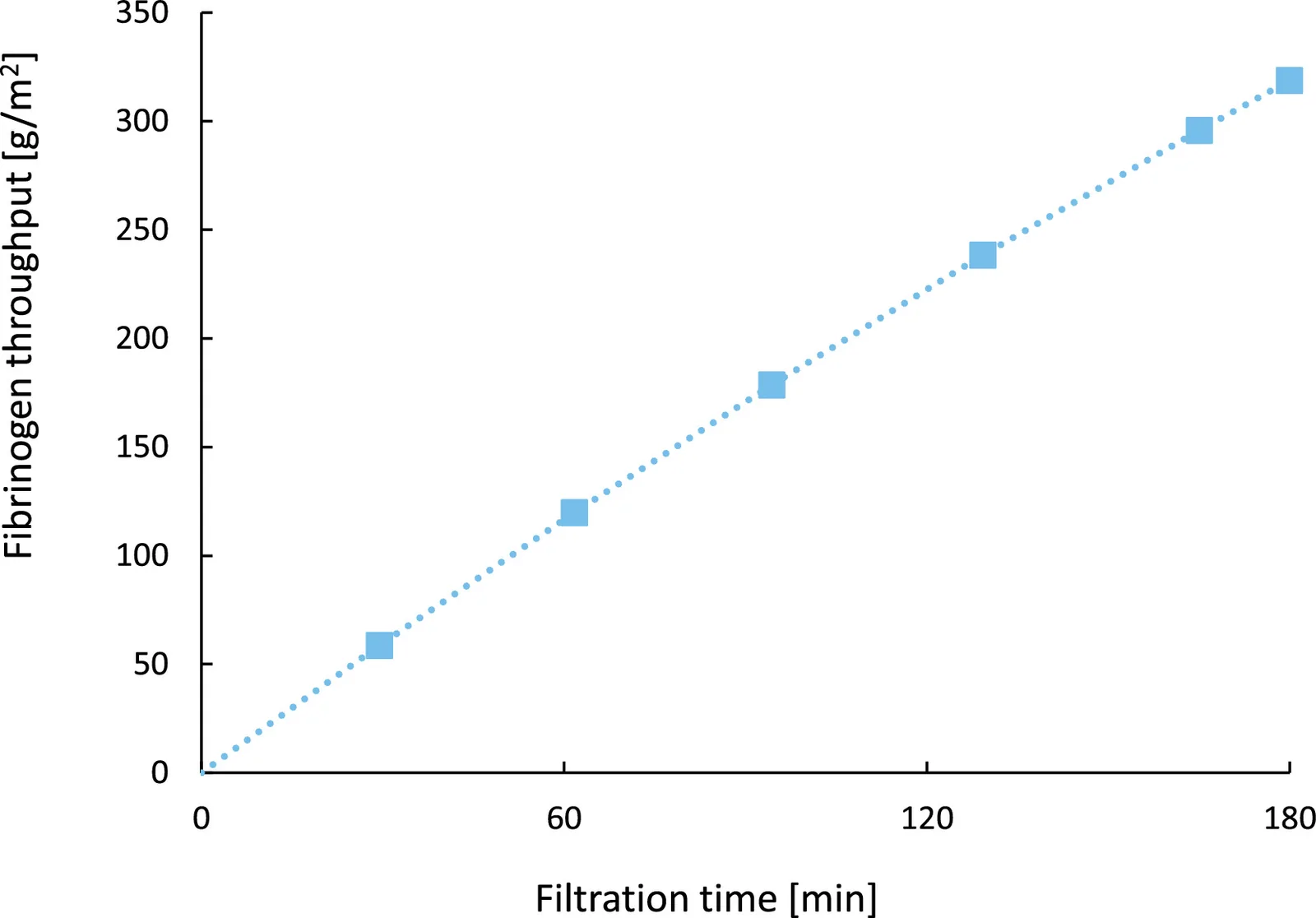
Fibrinogen throughput for filtration spiked with PPV.

**Fig. 6:**
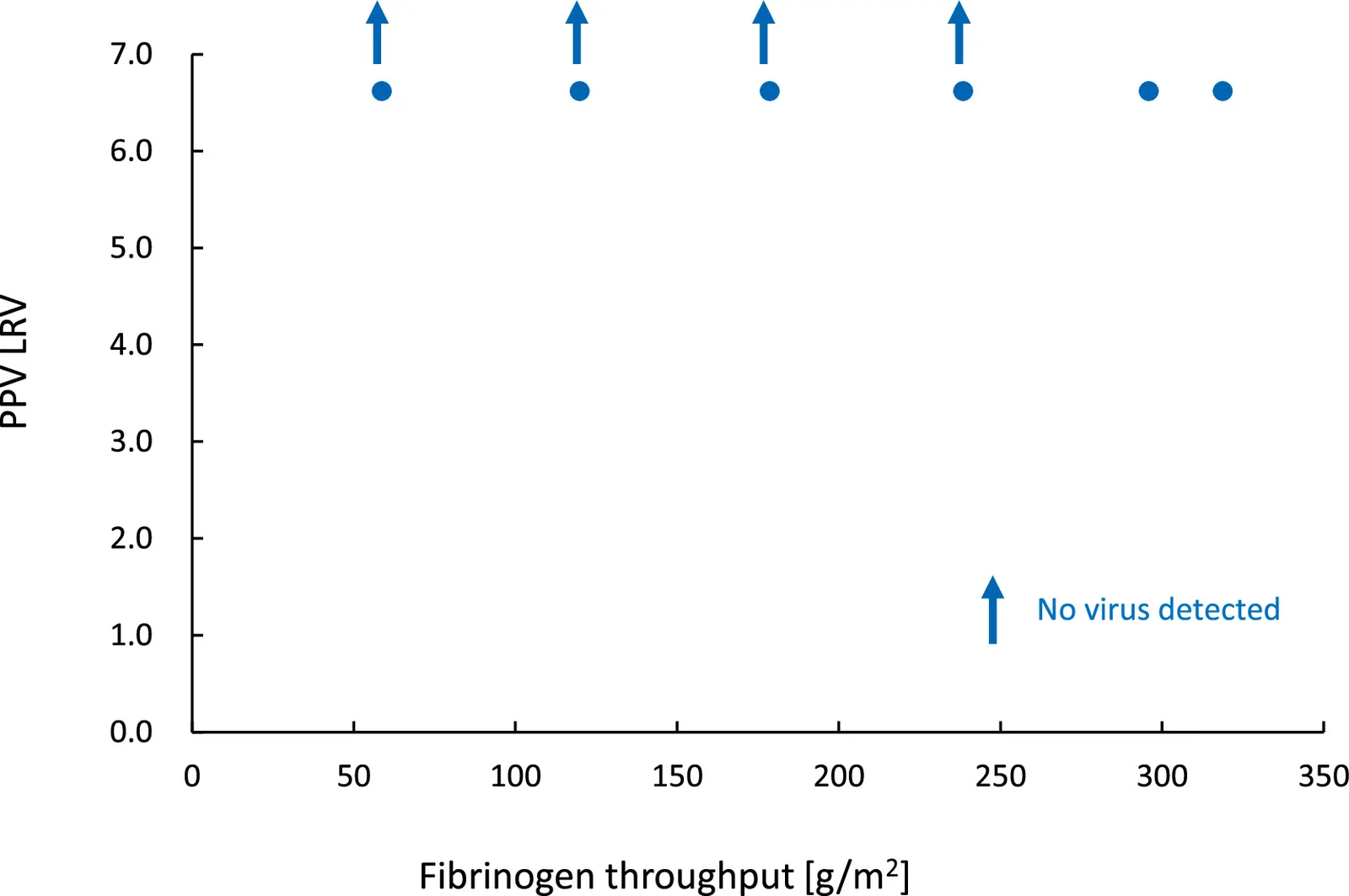
PPV LRV as a function of fibrinogen throughput.

For filtrations of 2 mg/mL fibrinogen spiked with PPV in 50 mM citric acid, 400 mM arginine, 150 mM NaCl, pH 7 with filtration at 35 °C, high fibrinogen throughput was achieved (Fig. 5). The PPV logarithmic reduction value (PPV LRV) for the filtration was 6.6 and or more (Fig. 6).

For filtrations of 2 mg/mL fibrinogen spiked with PPV in 50 mM citric acid, 400 mM arginine, 150 mM NaCl, pH 7 with filtration at 35 °C, high fibrinogen throughput was achieved (Fig. 5). As noted in the Methods section, individual PPV particles (∼20 nm) were not retained by the Planova 35 N pre-filter; accordingly, the PPV LRV measured in this experiment is attributable solely to the Planova 20 N virus removal filter. The PPV logarithmic reduction value (PPV LRV) for the Planova 20 N filtration step was ≥6.6 (Fig. 6).

## 4 Conclusions

The application of Planova 20 N virus removal filters to fibrinogen processes can enhance the virus safety of these preparations by effectively eliminating potential viral contaminants. To ensure efficient filtration of fibrinogen solutions, it is necessary to minimize the average particle size of fibrinogen in the solution as measured by DLS. This requires careful optimization of the following parameters: arginine addition, fibrinogen concentration, and pre-filtration. To further increase the filtration flux, elevating the filtration temperature is considered effective, as it reduces the viscosity of the solution. Optimization of these parameters contributes to improved solubility and reduced aggregation, thereby facilitating more efficient filtration. Further, pre-filtration prior to virus filtration is critical for enhancing filterability. Pre-filtration using Planova 35 N filters, which have a larger pore size distribution, is effective for removing particulate impurities and protecting the filtration performance of the subsequent virus removal step.

## Funding

No external funding was received for this work.

## CRediT authorship contribution statement

**Tomona Harada:** Writing – original draft, Methodology, Investigation, Data curation. **Kumiko Michikawa:** Writing – original draft, Methodology, Investigation, Data curation. **Mana Konno:** Investigation. **Takeshi Yokoyama:** Writing – review & editing, Data curation. **Mikihiro Yunoki:** Writing – review & editing. **Daisuke Miyamoto:** Writing – original draft, Project administration, Conceptualization.

## Declaration of competing interest

The authors declare that they have no known competing financial interests or personal relationships that could have appeared to influence the work reported in this paper.

## Data Availability

Data will be made available on request.
